# Bilateral Knee Dislocations Treated with Acute, Single-Stage Multiligament Reconstructions

**DOI:** 10.1155/2021/9985788

**Published:** 2021-05-03

**Authors:** Casey Imbergamo, Andrzej Brzezinski, Tiffany Smith, Patrick S. Buckley, Kenneth G. Swan

**Affiliations:** Rutgers - Robert Wood Johnson Medical School Department of Orthopaedic Surgery, USA

## Abstract

Bilateral knee dislocations are exceedingly rare in orthopaedics. Managing these injuries presents a difficult task given their high complication rate and guarded prognosis. We report the case of a 21-year-old male who presented to our institution with bilateral knee dislocations sustained in a motor vehicle collision. The patient subsequently underwent multiligament knee reconstruction surgeries for each knee at one and three weeks following the initial injury. At one-year follow-up, the patient has achieved a successful outcome and has returned to regular activities which include hiking and exercising at the gym.

## 1. Introduction

Knee dislocations represent an uncommon and complex injury with a reported incidence of less than 0.02% of all orthopaedic injuries [1]. Bilateral knee dislocations occur even less frequently than unilateral dislocations, and the literature on such injuries is limited [2]. Managing knee dislocations presents a difficult task due to a high incidence of complications such as recurrent instability, neurovascular injury, stiffness, future arthritis, meniscus and cartilage damage, acute compartment syndrome, and deep venous thrombosis [2, 3]. The prognosis after bilateral knee dislocation may be poor [4].

There is a paucity of literature regarding bilateral knee dislocations with most information being limited to case reports [5–7]. Due to the complex nature of these injuries and lack of established treatment guidelines, a definitive approach to management must be determined individually. The purpose of this article is to outline the management of a patient who presented to our institution with bilateral knee dislocations sustained in a motor vehicle collision. Informed consent for publication of this case report was obtained from the patient.

## 2. Case Presentation

**Figure 1:**
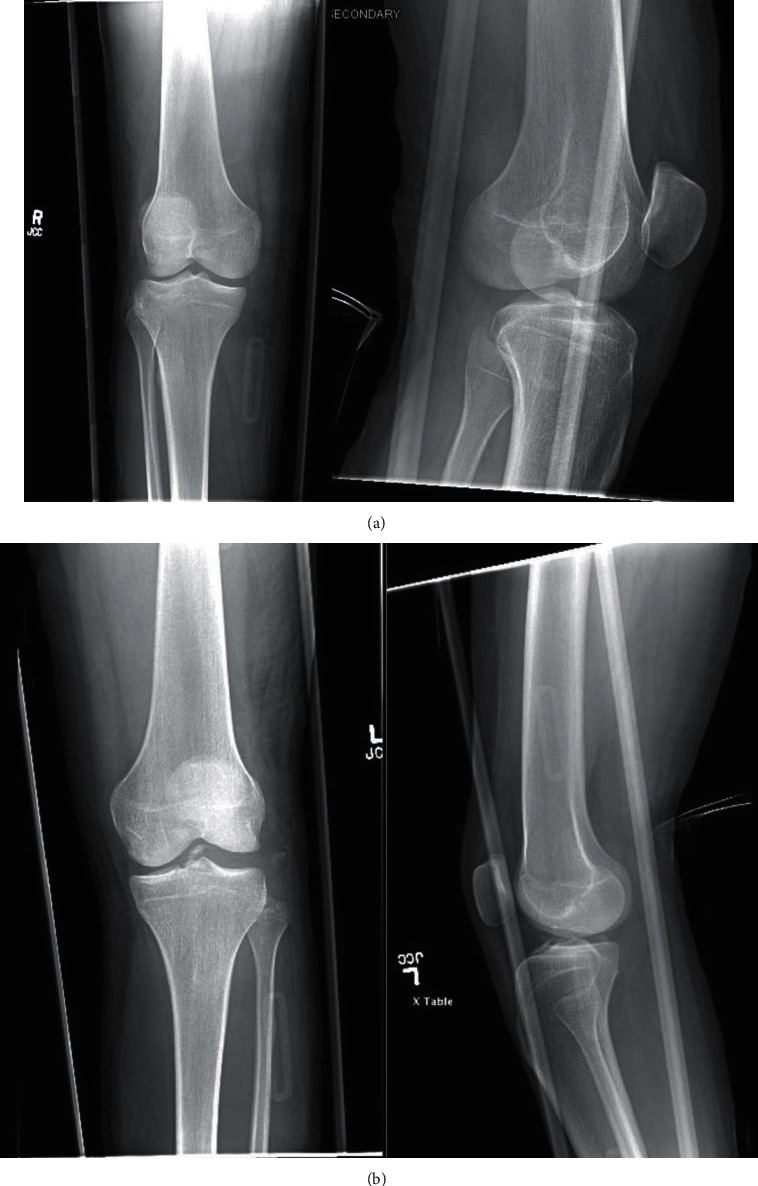
(a) Postreduction X-rays of the right knee. (b) Postreduction X-rays of the left knee.

**Figure 2:**
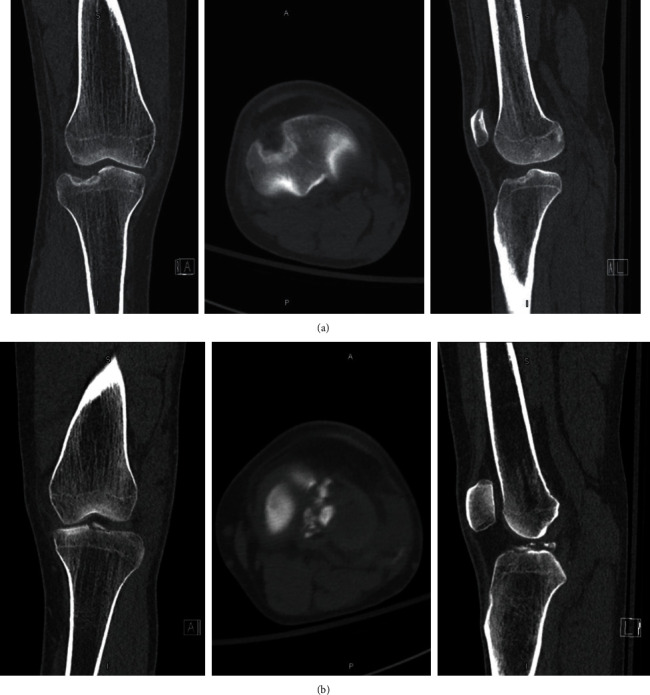
(a) CT scan of the right knee depicting a lateral tibial plateau fracture. (b) CT scan of the left knee depicting a tibial spine fracture.

**Figure 3:**
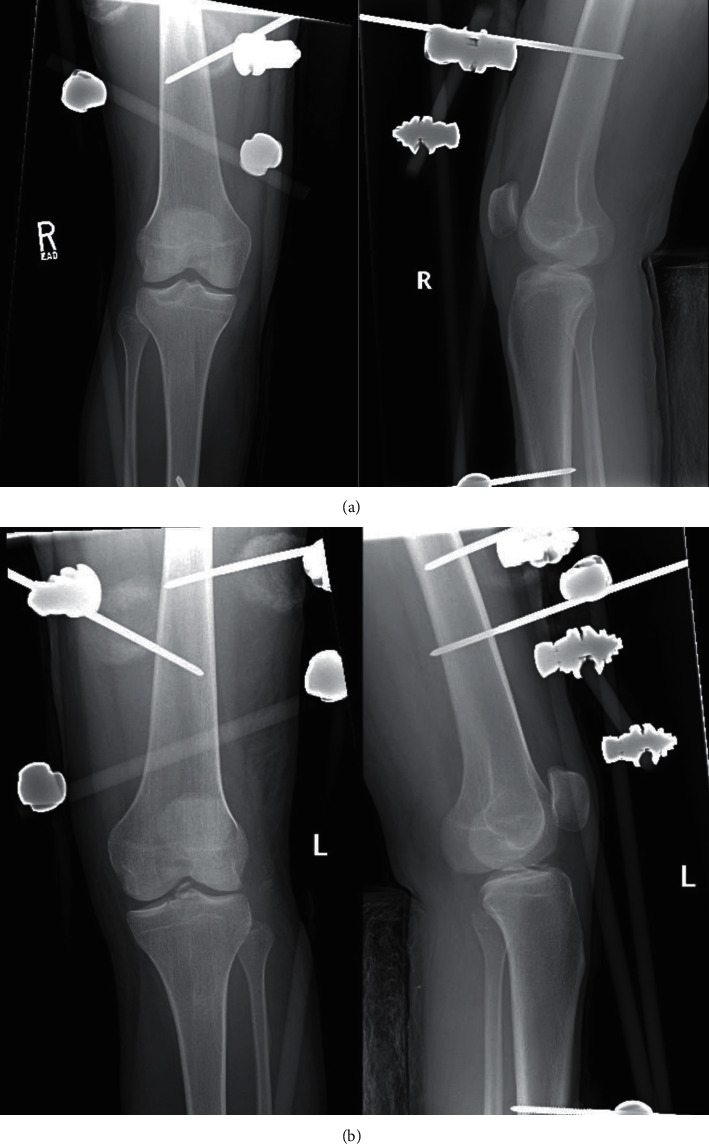
(a) External fixator application of the right knee. (b) External fixator application of the left knee.

The patient is a 21-year-old male who presented to our institution after being involved in a high-speed motor vehicle collision. He was the restrained driver, and his car rolled over and collided with a tree at highway speeds. The patient presented with isolated bilateral knee dislocations in a windswept pattern. Trauma workup performed at arrival revealed no other injuries. Within 90 minutes of injury, the patient underwent closed reduction of both knees with application of knee immobilizers. Given the obvious deformities of both knees, the bilateral knee dislocations were reduced prior to obtaining imaging, and X-rays were acquired postreduction. CT scans were also obtained to gain a better understanding of the bony involvement (Figures 1(a), 1(b), 2(a) and 2(b)). Postreduction evaluation revealed no vascular or neurologic injuries with soft compartments bilaterally. Ankle-brachial index (ABI) measurements evaluated pre- and postreduction were >0.9 bilaterally and were repeated for 48 hours to ensure no vascular changes presented in a delayed fashion. Due to gross instability of the knees following reduction, the decision was made to apply external fixators (Figures 3(a) and 3(b)).

**Figure 4:**
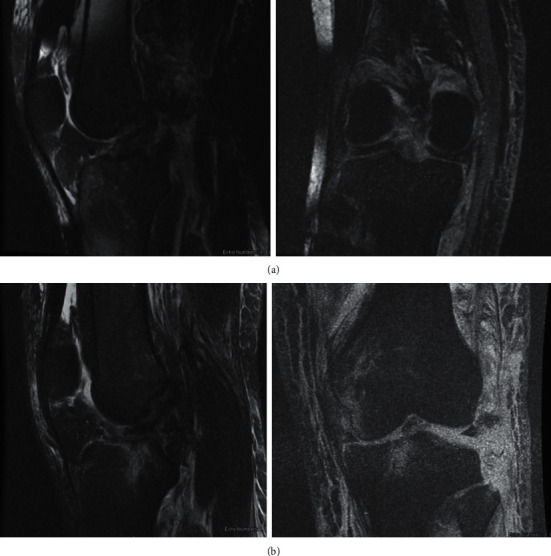
(a) MRI of the right knee depicting ACL and PCL rupture (left) and MCL rupture (right). (b) MRI of the left knee depicting ACL avulsion and PCL rupture (left) and LCL avulsion and PLC injury (right).

After initial stabilization, the decision was made to obtain an MRI to further evaluate the extent of soft tissue injuries. Even though the external fixator components were compatible with MRI, the patient complained of burning symptoms at the pin sites during initial attempt at obtaining the MRI. The decision was made to remove the external fixation devices, and the patient was subsequently placed in cylindrical casts to obtain MRI images. MRI was obtained and confirmed the injury patterns that were classified according to the anatomic classification of knee dislocation as a KD-V on the right and KD-IV on the left, respectively [8] (Table 1 and Figures 4(a) and 4(b)). The decision was made to proceed with bilateral knee multiligament reconstruction.

**Figure 5:**
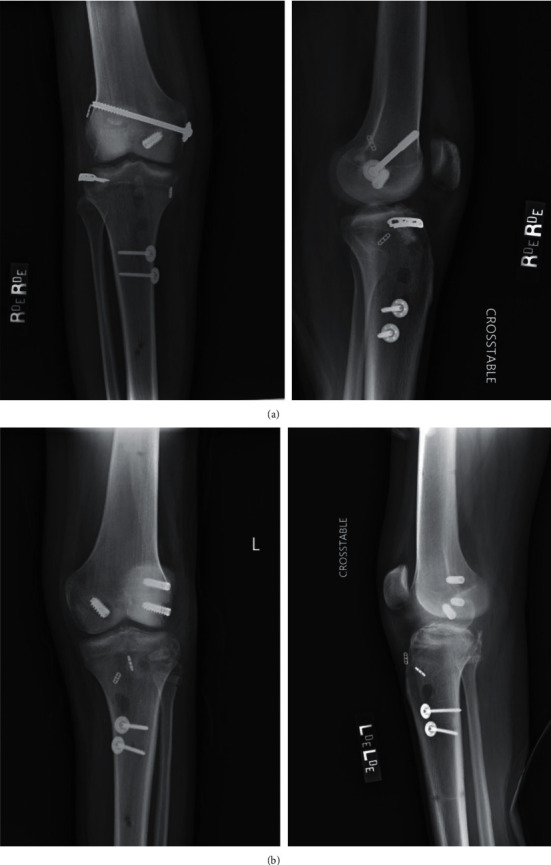
(a) Postoperative X-ray of the right knee. (b) Postoperative X-ray of the left knee.

The procedures were performed at one and three weeks from presentation, with the left knee being reconstructed first. A combined arthroscopic and open approach was utilized for both multiligament knee reconstructions (Table 2 and Figures 5(a) and 5(b)). Perioperatively, the patient received antibiotic prophylaxis with three doses of 2 g of Ancef and DVT prophylaxis with 30 mg of enoxaparin sodium administered every 12 hours for 6 weeks. Upon completion of each case, the operated knee was stable in both coronal and sagittal planes. Following each procedure, the respective knee was immobilized in a hinged knee brace in extension, and physical therapy was started at postoperative day one. The patient was briefly in an inpatient rehabilitation facility but opted to be discharged and received in-home therapy 1-2 times per week. The rehab protocol emphasized early prone range of motion from 0 to 90° for 2 weeks. The patient remained in a knee immobilizer for 2 weeks, was non-weight-bearing for 10 weeks, and then transitioned to weight-bearing as tolerated. Given the bilateral nature of the injuries, the patient remained non-weight-bearing for an extended period of time (10 weeks as opposed to 6 weeks), as he did not have a functioning contralateral leg to allow for full weight-bearing support.

The patient presented for initial outpatient follow-up four weeks following the injury. At this time, all wounds had healed appropriately with no erythema, drainage, or excessive swelling. The patient remained non-weight-bearing at this time and was utilizing a wheelchair. His pain was controlled with naproxen, acetaminophen, and Percocet 2-3 times per week. On exam, both knees were grossly stable; however, there was significant stiffness bilaterally. The right knee had passive range of motion (PROM) from 0 to 20°, and the left knee from 0 to 50°. The Knee Society Scores were 45 and 39 for the left and right knees, respectively [10]. The importance of physical therapy was emphasized to the patient at each follow-up visit, and he was made aware that an aggressive therapy regimen would be required in order to regain full motion of the knees. However, due to a multitude of factors, including the ongoing COVID-19 pandemic, the patient failed to progress through his rehabilitation protocol over the subsequent weeks.

Based on his inability to regain full range of motion with physical therapy, the patient required manipulation under anesthesia (MUA) of bilateral knees to improve range of motion. MUA was performed twice on the left side (week 2 at time of right-sided surgery and week 12 after reconstruction) and once on the right side (week 10 after reconstruction). Manipulation under anesthesia was implemented rather than lysis of adhesions, as it was felt to be a less invasive procedure that would convey similar benefits for the patient.

The patient continued in-home therapy. At the last MUA procedure, both knees were ultimately able to obtain full flexion and extension under anesthesia. Both knees remained stable after the procedure. The patient continued with physical therapy focusing on ROM, gait mechanics, and strengthening. He required oxycodone on physical therapy days; otherwise, his pain was controlled with acetaminophen and naproxen. At four-month follow-up, the left knee had active range of motion (AROM) from 0 to 130° and PROM from 0 to 135°. The right knee had AROM from 5 to 80° and PROM from 0 to 90°. At six-month follow-up, the left knee exhibited full AROM from 0 to 135°, and the right knee had advanced to an AROM of 0-115° and PROM of 0-125°.

At nine months from initial injury, the patient was able to walk unassisted without difficulty.

He was continuing physical therapy for range of motion and strengthening. A 5° flexion contracture of the right knee persisted. He had not yet begun running or returned to sport.

**Figure 6:**
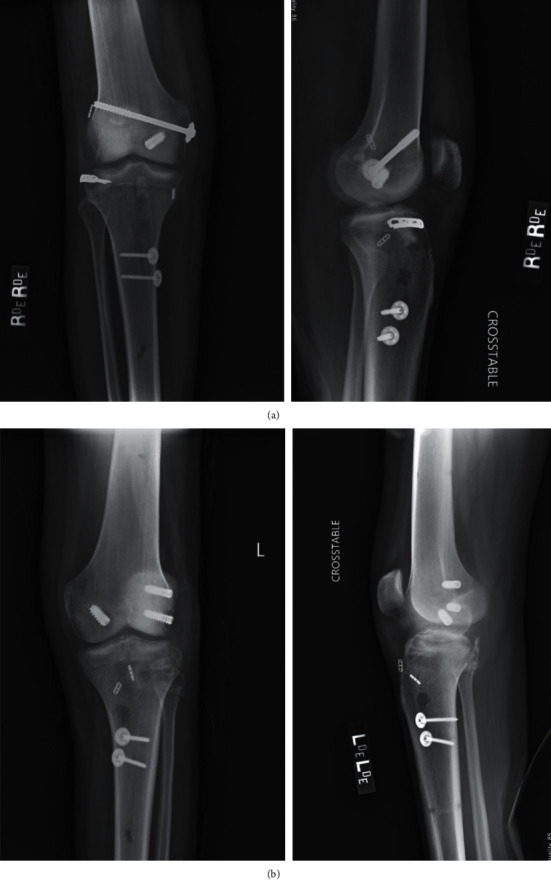
(a) Follow-up X-ray of the right knee. (b) Follow-up X-ray of the left knee.

At one-year follow-up, the Knee Society Scores were 90 and 89 for the left and right knees, which improved from 45 and 39, respectively [10]. The left knee demonstrated a full range of motion, while the right lacked the last 5° of flexion. All ligamentous repairs remained intact (Figures 6(a) and 6(b)). The patient was able to walk for an unlimited distance and navigate stairs without the use of assistive devices. He had returned to the gym for exercise and resumed his regular activities and hobbies. He reported occasional anterior knee pain brought on by sitting in deep knee flexion for extended periods of time, which was relieved with ibuprofen. Overall, the patient was satisfied with his outcome at one-year follow-up.

## 3. Discussion

Knee dislocations represent an uncommon and complex multiligamentous knee injury with a reported incidence of less than 0.02% of all orthopaedic injuries, with bilateral knee dislocations representing an even less frequent occurrence [1]. This case report outlined the management of a patient with bilateral knee dislocations with multiligamentous injuries who was able to achieve a successful outcome at one-year follow-up.

Bilateral knee dislocations present a difficult task in orthopaedic management. The prognosis of knee dislocations depends on the mechanism of the trauma, the amount of neurovascular damage, concomitant meniscus and chondral injuries, and the persistence of rehabilitation [6]. Despite a high complication rate following these injuries, it is possible to obtain satisfactory outcomes with the appropriate management focusing on the balance between stability and ROM of the involved knee [2, 6, 11, 12].

Controversy exists within the literature regarding the optimal treatment of these complex injuries; however, reconstruction with or without repair has been widely accepted over repair alone for ligamentous restoration [1, 13, 14]. Timing of repair is an area of debate; however, an acknowledgment of the benefits of concurrent anatomic reconstruction of all damaged ligaments is standard across the literature to allow for early postoperative knee range of motion [1]. There are three approaches to the timing of surgery for multiligamentous knee injury: acute, staged, or delayed [15].

Acute reconstruction, as was used in this case, is generally defined as surgery performed within three weeks of injury. This time frame is considered to be the critical in order to maintain soft tissue planes that are still definable without significant scarring [15]. This is especially important for planned posterolateral corner reconstruction with biceps femoris and/or IT band avulsion for easier peroneal nerve and lateral structure identification. Surgeons who advocate for acute surgery argue that by reconstructing all the damaged ligaments in an acute time frame enhances the chance of normal knee kinematics being restored [15]. In this case, the patient experienced bilateral knee stiffness which required MUA to improve ROM. However, it should be noted that the patient was inconsistent with his physical therapy regimen, and this may have contributed to the stiffness during the recovery period.

Another approach postulated by Colen et al. is a staged reconstruction of knee dislocations [6]. The staged reconstruction allows for initial healing of the articular capsule and collateral ligaments, especially the medial collateral ligament with postponed reconstruction of the intra-articular structures. This approach allows for gradual recovery from the injury; however, the recovery time is prolonged. The benefit from the staged approach may be avoiding intraoperative complications like acute compartment syndrome which can develop when a torn capsule is infiltrated with a significant amount of fluid during the arthroscopy [6]. However, this complication can be avoided by allowing the egress of fluid through the capsule by making open approaches first.

Hirschmann et al. evaluated the timing of surgical intervention after the injury. He found that complete single-stage reconstruction/primary repair conducted within 40 days from the injury correlated with good results and a high rate of return to the same level of activity as before trauma. Those who underwent surgery in delayed fashion (>40 days postinjury) were found to have unsatisfactory results and had lower rates of return to professional sport. A key finding was that soft tissue edema and limited range of motion may correlate with increased risk of arthrofibrosis, and initiation of surgery should be carried out early to optimize outcomes [11, 15].

The patient in our case underwent acute reconstruction with his left knee reconstruction occurring 6 days postinjury and reconstruction of his right knee 21 days postinjury. Throughout the patient's postoperative course, the ROM of his right knee was substantially deficient compared to the left knee. However, following bilateral MUA, the patient experienced significant improvement in range of motion of both the right and left knees. Similar to our case, Cottet et al. reported a case of bilateral reconstruction of both knees that required postop MUA. The patient in the case reported by Cottet et al. was able to return to partial weight-bearing by 6 weeks and full weight-bearing by 10 weeks. By 10-month postinjury, he was able to return to high-level activities, and by 20 months, he was asymptomatic and back to competing at world-class skiing championships [2]. This highlights the utility of MUA in the postreconstruction plan to achieve maximal ROM and limit stiffness if the patient is unable to progress in their rehabilitation protocol and advance ROM.

A study by LaPrade et al. reported results from a cohort of patients who underwent single-stage multiligament knee reconstructions and found that almost 10% of patients developed arthrofibrosis requiring reoperation [16]. This further highlights the utility of MUA as a modality to regain adequate ROM postoperatively. Manipulation under anesthesia should be considered in the routine postoperative care, and the patient should be educated about the possibility of a MUA prior to undergoing the initial procedure. This should not be considered a failure or complication of the procedure, rather an additional aspect of a comprehensive approach to treating these complex injuries.

In this case, each knee was immobilized in a hinged knee brace in extension following multiligament reconstruction. However, applying an articulated external fixator postoperatively can also be considered as a viable option. A study by Angelini et al. compared the use of rigid knee bracing versus articulated external fixator application following multiligament reconstruction [17]. The authors found that patients in the articulated external fixator group achieved significantly greater ROM with the same ligament stability, in addition to improved subjective measures of functional status [17]. As stiffness is a common complication following multiligament knee reconstruction, the application of a hinged external fixator may be considered as an option to improve outcomes, particularly ROM, in these patients.

The presented case is an example of a successful surgical reconstruction of multiligamentous injuries following bilateral knee dislocations. This case, in addition to previously published reports in the literature, represents the prospect of achieving a good outcome with return to function following these devastating injuries.

## 4. Conclusion

While bilateral knee dislocations present difficult orthopaedic injuries to manage, successful outcomes can be achieved with appropriate management. Acute reconstruction should be considered to optimize restoration of knee kinematics. Additionally, MUA can be implemented in the recovery period to maximize range of motion. In this case of a 21-year-old male who suffered bilateral knee dislocations, implementation of these techniques yielded a successful outcome at one-year follow-up.

## Figures and Tables

**Table 1:** List of injuries sustained.

Summary of injuries sustained to each knee	
*Right knee*	*Left knee*
Lateral tibial plateau fracture (Schatzker III)	ACL avulsion from tibia
ACL rupture	PCL rupture
PCL rupture	PLC injury
MCL rupture	LCL avulsion from fibula
PMC disruption	Lateral meniscal root tear
Medial meniscal root tear	Biceps femoris avulsion from fibula
	Iliotibial band tear (at joint line)

ACL: anterior cruciate ligament; PCL: posterior cruciate ligament; MCL: medial collateral ligament; PMC: posteromedial corner; PLC: posterolateral corner; LCL: lateral collateral ligament.

**Table 2:** Procedures performed on each knee.

Reconstruction performed	Graft/plate	Fixation
*Right knee*		
Tibial plateau ORIF	Unicortical locking plate	3 unicortical locked screws
ACL reconstruction	Quadriceps tendon autograft	Femoral: EndoButton (Smith & Nephew), tibial: Regenesorb screw (Smith & Nephew)
PCL reconstruction (double bundle)	Split Achilles tendon allograft	Femoral: Regenesorb screw (Smith & Nephew) (PM bundle), metallic interference screw (AL bundle), tibial: 4.5 mm bicortical screws with spiked washers
MCL reconstruction	Semitendinosus autograft	Femoral: cannulated partially threaded screw with spiked washer, tibial: Q-Fix anchor (Smith & Nephew)
Posteromedial corner reconstruction	Semitendinosus autograft	Femoral: cannulated partially threaded screw with spiked washer
Medial meniscal root repair (two-tunnel)		4-hole cortical button
*Left knee*		
PCL reconstruction	Split Achilles allograft	Femoral: Regenesorb screw (Smith & Nephew) (PM bundle), metallic interference screw (AL bundle), tibial: 4.5 mm bicortical screws with spiked washers
PLC reconstruction (two-tailed LaPrade reconstruction of FCL and popliteus) [9]	Split Achilles allograft	Femoral: interference screws proximally, Regenesorb screws (Smith & Nephew) 1 in tibia, 1 in fibula
ACL avulsion repair		UltraTape sutures (Smith & Nephew) with EndoButton (Smith & Nephew)
Lateral meniscal root repair (single tunnel)		UltraTape sutures (Smith & Nephew) with EndoButton (Smith & Nephew)
Biceps femoris avulsion repair		HealiCoil Anchor in fibula (Smith & Nephew)
Iliotibial band repair		Q-Fix Anchor in distal femur (Smith & Nephew)

ORIF: open reduction internal fixation; ACL: anterior cruciate ligament; PCL: posterior cruciate ligament; PLC: posterolateral corner.
